# Improving mental health in black men through a 24-week community-based lifestyle change intervention: the black impact program

**DOI:** 10.1186/s12888-023-05064-5

**Published:** 2024-01-09

**Authors:** Joshua J. Joseph, Timiya S. Nolan, Guy Brock, Amaris Williams, Songzhu Zhao, Alicia McKoy, Bjorn Kluwe, Faith Metlock, Katherine Campanelli, James B. Odei, Monique T. Khumalo, Dana Lavender, John Gregory, Darrell M. Gray

**Affiliations:** 1 The Ohio State University College of Medicine, Suite 5000, 700 Ackerman Road, Columbus, OH 43202 USA; 2 The Ohio State University College of Nursing, Columbus, OH USA; 3 The Ohio State University James Center for Cancer Health Equity, Columbus, OH USA; 4 The Ohio State University College of Public Health, Columbus, OH USA; 5 University of Kentucky, Lexington, KY USA; 6 The African American Male Wellness Agency, Columbus, OH USA; 7 National Center for Urban Solutions, Columbus, OH USA

**Keywords:** Black Men, Mental health, Depression, Stress, Life’s simple 7, Community-based participatory research, Cardiovascular disease, Health equity

## Abstract

**Background:**

Poor mental health is a leading cause of morbidity and mortality among Black men in the United States. Efforts to improve mental health among Black men have been hampered by a lack of access and utilization of mental health services. Physical activity and social networks have been shown to improve mental health. Thus, we examined the effect of a community team-based physical activity, health education and social needs intervention among Black men on mental health over 24 weeks.

**Methods:**

Black adult males (n = 74) from a large Midwestern city participated in Black Impact, a 24-week community-based lifestyle change program adapted from the Diabetes Prevention Program and American Heart Association’s (AHA) Check, Change, Control Blood Pressure Self-Management Program, which incorporates AHA’s Life’s Simple 7 (LS7) framework. Measures of mental health including the Center for Epidemiological Studies Depression Scale (CES-D), Patient Health Questionnaire 2-question depression screener (PHQ-2), and Perceived Stress Scale-10 (PSS-10) were completed at baseline, 12 and 24 weeks. The change in mental health scores from baseline to 12 and 24 weeks were evaluated using linear mixed-effects models adjusting for age, education, and income. The change in cardiovascular health scores, defined as objective metrics of LS7 (LS5 [blood pressure, total cholesterol, fasting glucose, body mass index and smoking]), by baseline mental health were evaluated using linear mixed-effects models with an interaction term (time*baseline mental health variable) and a random intercept for each participant.

**Results:**

Among 71 Black men (mean age 51, 85% employed) at 24 weeks, CES-D scores decreased from 10.54 to 7.90 (-2.64, 95%CI:-4.74, -0.55), PHQ-2 decreased from 1.04 to 0.63 (-0.41, 95%CI: -0.75, -0.07), and PSS-10 decreased from 14.62 to 12.91 (-1.71, 95%CI: -3.53, 0.12). A 1-unit higher CES-D at baseline was associated with less improvement in LS5 scores by -0.04 (95%CI: -0.076, -0.005) and − 0.032 (95%CI:-0.067, 0.003) units at week 12 and 24, respectively, with similar findings for PSS.

**Conclusions:**

The Black Impact community-based lifestyle program has the potential to reduce depressive symptoms and stress in Black men. There is a dire need for larger, randomized studies to test the impact of Black Impact on mental health in Black men to advance health equity.

**Trial Registration:**

Retrospectively Registered, ClinicalTrials.gov Identifier: NCT04787978.

**Supplementary Information:**

The online version contains supplementary material available at 10.1186/s12888-023-05064-5.

## Introduction

Mental health disorders are a leading cause of morbidity in the United States, with nearly one in five adults living with a mental health disorder (mental, behavioral, or emotional disorder in the past year of sufficient duration to meet DSM-IV criteria [excluding developmental disorders and substance use disorders]) in 2021 [1]. 17% of Black Americans self-reported a mental health disorder in 2020 [2]. Rates of major depression are increasing in Black adults, who are more likely than White adults to experience persistent symptoms of emotional distress, such as sadness, hopelessness, and feeling that they have to dedicate extra effort to everything they do [3]. Currently, there exists a serious underutilization of mental health services including outpatient mental health visits and prescription psychiatric medications, among Black Americans, particularly Black men [4, 5]. The underutilization of services, care and medications is made worse by the lack of Black mental health providers. Black providers only represent 3% of total psychiatry faculty [6]. In 2015, only 4% of psychologists were Black [7]. The lack of Black mental health providers is key barrier to effective care, as client-rated measures of therapist cultural competence correlate strongly with treatment outcomes [8]. Cultural adaptations to mental health treatments typically prove more effective than treatment as usual with clients of color in North America [8]. The deficit in the knowledge and skills in treating depression in Black Americans, in addition to inadequate and insufficient data on Black Americans, contributes to the challenges of under diagnoses, misdiagnosis, and under treatment of depression and is a product of structural racism [9, 10].

Mental health has significant implications for cardiovascular health in Black Americans. Depression [11, 12] and perceived psychosocial stress [13–15], in particular, are associated with increased risk of cardiovascular disease and the combined effects of depression and perceived psychosocial stress may be even more ominous [16, 17].

Since the establishment of the American Heart Association’s Life’s Simple 7 (LS7) framework [18], which identified 7 factors correlated with cardiovascular health – three health behaviors (diet, smoking, and physical activity) and four biometric measures (body mass index, blood pressure, blood glucose and cholesterol) – analyses have suggested a graded association of depression and/or stress with worse cardiovascular health defined by LS7 and vice versa, with a stronger relationship with behavioral factors [19]. However, there remains a paucity of evidence examining the association of mental health with LS7 specific to community-dwelling Black men or interventions to address mental health in the context of LS7. This is particularly troubling, given that Black men have the lowest attainment of cardiovascular health [20], shortest life-expectancy of any race/sex group [21], and a lack of community-based participatory research interventions aimed at improving LS7 [22]. Community-based participatory research was created to advance health equity and promote community empowerment in marginalized communities, while applying scientific rigor and principles [23]. Community-based participatory research is focused on developing collaborative partnerships facilitating equal input from the community and its stakeholders throughout planning, implementation, evaluation, and dissemination of research [23].

With the goal of reducing premature death from chronic disease and improving holistic health, the African American Male Wellness Agency (AAMWA) was founded in 2004, initiating multiple initiatives and partnerships that led to evaluating and examining LS7 cardiovascular health [24–26]. Given the poor levels of cardiovascular health at African American Male Wellness Walks [24], clinician-scientists from The Ohio State University co-designed and implemented a pilot 24-week community-based lifestyle intervention, Black Impact, focused on health education, physical activity and addressing non-medical health-related social needs, in partnership with AAMWA and many community organizations with the aim of improving the attainment of LS7 in Black men living in a large Midwestern city [27]. Black Impact improved LS7 scores at weeks 12 and 24 compared to baseline [27].

Black Impact included components that may impact mental health including sessions discussing mental health topics, weekly physical activity [28, 29] and organic development of social networks [30–33]. Given the importance of examining and addressing the multi-faceted harms from poor mental health ranging from direct consequences such as suicide to worse cardiovascular health and cardiovascular outcomes [19, 34–41], this manuscript examines the effect of Black Impact on depressive symptoms and perceived stress among community-dwelling Black men. The authors hypothesized improvements in mental health from baseline to 12 and 24 weeks and an association between baseline and change in mental health with attainment of cardiovascular health over the 24-week intervention.

## Methods

### Study design and recruitment

The Black Impact community-based participatory research intervention has been described previously [27]. Briefly, in this single-arm pilot program, we enrolled Black men from the annual AAMWA walk/health fair with poor or average cardiovascular health (< 4 LS7 metrics in the ideal range). The inclusion criteria included: (1) Black men (self-report); (2) adults aged 18 years or older; (3) English speaking; (4) live in Metropolitan Columbus, Ohio area; (5) no healthcare provider-imposed limitations on physical activity; and (6) participant is appropriate for a group setting (e.g., does not have untreated psychosis or behavioral challenges). In July 2020, the study began with 74 participants and the programming phase was implemented over 24 weeks through December 2020. The flow of participants through the intervention is shown in Supplemental Fig. 1. The sample size was based on the number of participants needed to determine effect sizes for the primary outcome (50–100 participants). Baseline, 12 and 24-week biometric health screenings occurred at study sites, and survey data were collected electronically via research electronic data capture (REDCap). The study was reviewed and approved by The Ohio State University Biomedical Sciences Institutional Review Board (Study ID: 2019H0302). The study was retrospectively registered on ClinicalTrials.gov: NCT04787978 (09/03/2021). All participants provided written informed consent.

### Intervention

The 24-week community-based lifestyle intervention aimed to improve cardiovascular health among Black men. The intervention was adapted from the Diabetes Prevention Program [42] and American Heart Association Check, Change, Control programs applying evidence-based strategies and stakeholder feedback [43]. Thus, participants were not randomized, and all received the entire intervention. Each participant was assigned to a community health worker and grouped into 6 teams of 8–25 participants based on participant proximity to a central meeting location (e.g. Columbus Recreation and Parks recreation center). Each team had a personal trainer who delivered the physical activity curriculum. The personal trainers had pre-intervention meetings with American College of Sports Medicine Certified Personal Trainer (ACSM-CPT). They were trained in a standardized, 45-minute workout with increasing intensity over the study intervention consistent with Exercise is Medicine [27].

The 30-minute health coach led sessions focused on the Diabetes Prevention Program, AHA Check, Change, Control Curriculum, cooking, grocery store shopping, mental health, historical trauma, stress, financial wellness, and cancer screening [27]. Specifically in relation to mental health in Week 8, historical trauma, stress management and mental health strategies & resources were discussed by a mental health counselor from a local public health agency (18 min), followed by an introduction to a local resource for Black men to discuss mental health on a quarterly basis in a large group format 100–200 men (AAMWA Barbershop Talk and Real Men, Real Talk, 2 min) and DPP Session 15: Stress Management (10 min). In Week 13, DPP Session 11: Talk Back to Negative Thoughts (30 min) has mental health components [44]. Fourteen participants in the program attended one session of Barbershop Talk: Real Men, Real Talk where they discussed the importance of Black men being able to talk about mental health issues (2 h). Key health coach activities included delivering education and establishing and monitoring progress in achieving individual and team-based SMART (specific, measurable, achievable, relevant, and time-bound) wellness goals. The health coaches were healthcare providers (2 physicians and 1 nurse practitioner) experienced in lifestyle change, each assigned 2 teams. They were assisted by Ohio State University nursing students from Nursing Students Promoting Initiatives Reinforcing Equity (NSPIRE). All instructors were trained in the curriculum and protocol. Healthy food samples, cross-trainer shoes, GARMIN watches, and workout bands were provided to all participants. Individual incentives (e.g., gift cards) were provided to participants for follow-up survey completion. All participants received a one-year gym membership to a local recreation and park center at the study end for participating in the intervention.

### Data collection and measures

Assessments were performed at baseline, 12 weeks, and 24 weeks. Data from participants included self-reported measures (sociodemographic and self-reported health history) and survey data collected via REDCap [27, 45]. The sociodemographic data included age, education, race, ethnicity, employment status, insurance status, and annual income. The self-reported health history included hypertension, diabetes, hyperlipidemia, and smoking status (I have never smoked, I currently smoke, I quit smoking > 1 year ago or I quit smoking ≤ 1 year ago), as well as medications for the aforementioned chronic conditions [27, 45].

The survey data included the following mental health questionnaires: Center for Epidemiological Studies Depression (CES-D) Scale,[46] Patient Health Questionnaire 2-question (PHQ-2) depression screener [47], Perceived Stress Scale (PSS) [48], and the Short Form Survey (SF-36) [49].

The CES-D survey is a 20-item scale measuring self-reported symptoms of depression experienced in the past week including depressed mood, feelings of guilt and worthlessness, feeling of helplessness and hopelessness, loss of energy, sleep disturbance, and change in appetite. Each item is scored 0 to 3 on a Likert scale (e.g. 0: “not at all”; 1: “a little”; 2: “some;” 3: “a lot”) for frequency of symptoms in the last week, for a total score range of 0 to 60 with higher scores suggesting a greater presence of depressive symptoms. The CES-D is validated across age, sex and race categories [50].

The PHQ-2 is a two-item questionnaire that assesses the frequency of depressed mood and anhedonia over the past two weeks [47]. Each question is scored on a ranges from 0 to 3 (0: “not at all;” 1: “several days;” 2: “more than one-half the days;” 3: “nearly every day”).

The PSS-10 is a 10 question stress assessment instrument to measure the perception of stress [48]. The questions in this scale ask about your feelings and thoughts during the last month and are scored on a 5-point Likert scale (0: “never;” 1: “almost;” 2: “sometimes;” 3: “fairly often;” 4: “very often”) [48]. The scale is valid and reliable [51].

The Short Form Survey (SF-36) is a 36-item questionnaire assessing self-reported health status. It is a general measure of health-related quality of life [52, 53]. Reliability estimates for summary measures are ≥0.90 [52, 53]. The SF-36 is composed of 8 multi-item scales (35 items) assessing physical function (10 items), role limitations due to physical health problems (4 items), bodily pain (2 items), general health (5 items), vitality (4 items), social functioning (2 items), role limitations due to emotional problems (3 items) and emotional well-being (5 items). These eight scales are aggregated into two summary measures: the Physical and Mental Component Summary (MCS) scores, as mental health symptoms increase, MCS scores decrease.

Biometric screenings including blood pressure (mmHg), fasting cholesterol (mmol/L), fasting glucose (mmol/L), weight (lbs), and BMI were performed by trained healthcare staff, including nurses and physicians. Blood pressure was checked via an automated oscillometric sphygmomanometer (Omron 5 series) with two measurements performed after the participants were seated for 5 min and averaged [27, 45]. Weight was measured using a zeroed and calibrated Omron Body Composition Monitor and Scale (Model: HBF-514 C). Height was measured via a tape measurer [27, 45]. BMI was calculated by multiplying weight (lbs) by 703 and then dividing by height squared (inch^2^). Blood total cholesterol and glucose were measured in the fasting state using the Cardio Check Silver® (Polymer Technology, Inc., Heath, OH, USA) device [27, 45].

A cardiovascular health (CVH) score was summed based on the individual LS7 metrics (glucose, cholesterol, blood pressure, BMI, physical activity, diet and smoking) categories of poor (0 points), intermediate (1) and ideal (2) CVH with a total score ranging from 0 to 14 at baseline, 12 and 24 weeks. Additionally, analyses used 6 components of the CVH score excluding diet (range 0–12; LS6) and 5 components excluding diet and physical activity (range 0–10; LS5), as has been done previously [27].

### Statistical analysis

Descriptive statistics were performed for all variables, including mean (standard deviation [SD]) for continuous variables and frequencies and percentages for categorical variables. Correlations between baseline variables were assessed via linear models in a pairwise fashion to evaluate concordance between the mental health measures. The primary analysis was change in mental health measures during the intervention. The change in mental health scores from baseline to 12 and 24 weeks were evaluated using linear mixed-effects models adjusting for age, education, and income. Secondary analyses included: (1) Change in CVH scores by baseline mental health were evaluated using linear mixed-effects models with an interaction term (time*baseline mental health variable) and a random intercept for each participant; and (2) The correlation between change in mental health and change in cardiovascular health scores by: (A) Fitting a mixed effect model describing each outcome variable with a random slope for time for each participant. These random slopes were extracted and saved. Linear models were run comparing the generated slopes for one outcome variable to the generated slopes from another outcome variable; and (B) Calculating differences (ΔX = X_1_-X_0_), where X_0_ is the baseline value and X_1_ is the value at either 12 or 24 weeks. Differences in one variable were used to describe differences in another variable in linear models. Statistical significance for all analyses was defined as two-sided alpha < 0.05. All statistical analyses were performed using R statistical software version 4.05 (R Foundation for Statistical Computing, Vienna, Austria).

## Results

The baseline demographics of 71 of the 74 Black Impact participants with data on PHQ-2 at baseline are shown in Table 1. The mean age of participants was 52 years (standard deviation [SD] 10.5). All participants had a high school degree or equivalent and 85% were employed, 73% had private insurance. The income of participants was diverse, ranging from <$20,000 (6%) to ≥$75,000 (23%). At baseline the mean CES-D was 10.9 (SD 8.98), 25.4% of participants had a CES-D ≥16, indicative of potential depression. The mean PHQ-2, PSS and SF-36 MCS were 0.99 (SD 1.50), 14.5 (SD 7.28) and 45.4 (SD 15.3), respectively. There was no difference in most sociodemographic measures and baseline CVH scores across PHQ-2 categories of 0 vs. ≥1. However, there was a difference in the AHA categorization of physical activity with higher levels among individuals with a PHQ-2 score of 0 vs. 1+ (p = 0.016). Significant correlation existed between baseline mental health measures, but there was no correlation of baseline mental health measures with baseline CVH scores in unadjusted linear models in Supplemental Tables 1 and negligible Pearson correlations in Supplemental Table 2.

**Table 1:** Characteristics of Participants who answered CMS PHQ-2 survey in Black Impact Pilot Study stratified by PHQ-2 Score 0 vs. 1+

	Overall (N = 71)	PHQ-2 Score 0 (N = 42)	PHQ-2 Score 1+ (N = 29)	p-value
**Age**	52.0 (10.5)	53.0 (10.2)	50.7 (10.9)	0.363
**Marital Status**				0.462
Married	38 (53.5%)	25 (59.5%)	13 (44.8%)	
Widowed	1 (1.4%)	1 (2.4%)	0 (0%)	
Divorced	13 (18.3%)	6 (14.3%)	7 (24.1%)	
Separated	1 (1.4%)	1 (2.4%)	0 (0%)	
Never Married	18 (25.4%)	9 (21.4%)	9 (31.0%)	
**Number of Children**	3.03 (1.56)	3.26 (1.52)	2.69 (1.58)	0.129
**Annual Income**				0.187
<$20,000	4 (5.6%)	4 (9.5%)	0 (0%)	
$20,000-$49,999	19 (26.8%)	7 (16.7%)	12 (41.4%)	
$50,000-$74,999	22 (31.0%)	11 (26.2%)	11 (37.9%)	
>= $75,000	16 (22.5%)	12 (28.6%)	4 (13.8%)	
Missing	10 (14.1%)	8 (19.0%)	2 (6.9%)	
**Employment Status**				0.236
Employed	60 (84.5%)	33 (78.6%)	27 (93.1%)	
Retired	7 (9.9%)	6 (14.3%)	1 (3.4%)	
Unemployed	4 (5.6%)	3 (7.1%)	1 (3.4%)	
**Education**				0.518
High School or equivalent	7 (9.9%)	6 (14.3%)	1 (3.4%)	
Some College	27 (38.0%)	13 (31.0%)	14 (48.3%)	
Vocational/Technical School (2 year)	7 (9.9%)	4 (9.5%)	3 (10.3%)	
College Graduate (4 year)	18 (25.4%)	11 (26.2%)	7 (24.1%)	
Master’s Degree (MS)	11 (15.5%)	7 (16.7%)	4 (13.8%)	
Professional Degree (MD,JD, etc.)	1 (1.4%)	1 (2.4%)	0 (0%)	
**Health Insurance Status**				0.480
Private insurance	52 (73.2%)	32 (76.2%)	20 (69.0%)	
Medicaid/Medicare	6 (8.5%)	3 (7.1%)	3 (10.3%)	
Military insurance	4 (5.6%)	1 (2.4%)	3 (10.3%)	
No insurance	9 (12.7%)	6 (14.3%)	3 (10.3%)	
**Systolic Blood Pressure (mmHg)**	139 (20.1)	139 (18.0)	140 (23.2)	0.943
Missing	1 (1.4%)	0 (0%)	1 (3.4%)	
**Diastolic Blood Pressure (mmHg)**	87.7 (13.4)	87.3 (11.6)	88.3 (16.0)	0.749
Missing	1 (1.4%)	0 (0%)	1 (3.4%)	
**Blood Glucose (mmol/L)**	6.9 (3.0)	7.3 (3.6)	6.4 (2.0)	0.197
**Total Cholesterol (mmol/L)**	4.1 (1.1)	4.2 (1.3)	4.0 (0.9)	0.555
**Body Weight (pounds)**	238 (64.8)	253 (70.4)	216 (48.9)	**0.017**
**Body Mass Index (kg/m2)**	33.2 (7.52)	34.8 (7.81)	30.9 (6.54)	**0.030**
**High Cholesterol Medication**				0.218
Yes	19 (26.8%)	14 (33.3%)	5 (17.2%)	
No	52 (73.2%)	28 (66.7%)	24 (82.8%)	
**Diabetes Medication**				0.304
Yes	18 (25.4%)	13 (31.0%)	5 (17.2%)	
No	53 (74.6%)	29 (69.0%)	24 (82.8%)	
**High Blood Pressure Medication**				0.921
Yes	36 (50.7%)	22 (52.4%)	14 (48.3%)	
No	35 (49.3%)	20 (47.6%)	15 (51.7%)	
**Life’s Simple 7 Body Mass Index**				0.057
Ideal	7 (9.9%)	3 (7.1%)	4 (13.8%)	
Intermediate	25 (35.2%)	11 (26.2%)	14 (48.3%)	
Poor	39 (54.9%)	28 (66.7%)	11 (37.9%)	
**Life’s Simple 7 Physical Activity** ^a^				**0.016**
Ideal	37 (52.1%)	25 (59.5%)	12 (41.4%)	
Intermediate	29 (40.8%)	17 (40.5%)	12 (41.4%)	
Poor	5 (7.0%)	0 (0%)	5 (17.2%)	
**Life’s Simple 7 Blood Glucose**				0.294
Ideal	18 (25.4%)	11 (26.2%)	7 (24.1%)	
Intermediate	32 (45.1%)	16 (38.1%)	16 (55.2%)	
Poor	21 (29.6%)	15 (35.7%)	6 (20.7%)	
**Life’s Simple 7 Blood Pressure**				0.162
Ideal	5 (7.0%)	1 (2.4%)	4 (13.8%)	
Intermediate	31 (43.7%)	20 (47.6%)	11 (37.9%)	
Poor	34 (47.9%)	21 (50.0%)	13 (44.8%)	
Missing	1 (1.4%)	0 (0%)	1 (3.4%)	
**Life’s Simple 7 Smoking**				0.387
Ideal	59 (83.1%)	37 (88.1%)	22 (75.9%)	
Intermediate	2 (2.8%)	1 (2.4%)	1 (3.4%)	
Poor	10 (14.1%)	4 (9.5%)	6 (20.7%)	
**Life’s Simple 7 Cholesterol**				0.072
Ideal	40 (56.3%)	20 (47.6%)	20 (69.0%)	
Intermediate	26 (36.6%)	17 (40.5%)	9 (31.0%)	
Poor	5 (7.0%)	5 (11.9%)	0 (0%)	
**Life’s Simple 7 Diet**				0.395
Ideal	1 (1.4%)	0 (0%)	1 (3.4%)	
Intermediate	32 (45.1%)	20 (47.6%)	12 (41.4%)	
Poor	28 (39.4%)	15 (35.7%)	13 (44.8%)	
Missing	10 (14.1%)	7 (16.7%)	3 (10.3%)	
**Physical Activity Minutes/Week** ^a^	225 (219)	253 (239)	184 (184)	0.197
**Life’s Simple 7 Score**	7.48 (1.76)	7.29 (1.67)	7.76 (1.88)	0.308
Missing	11 (15.5%)	7 (16.7%)	4 (13.8%)	
**CES-D**	10.9 (8.98)	6.17 (4.82)	17.6 (9.28)	**< 0.001**
Missing	1 (1.4%)	1 (2.4%)	0 (0%)	
**CES-D > 16**				**< 0.001**
Yes	18 (25.4%)	2 (4.8%)	16 (55.2%)	
No	52 (73.2%)	39 (92.9%)	13 (44.8%)	
Missing	1 (1.4%)	1 (2.4%)	0 (0%)	
**Perceived Stress Score**	14.5 (7.28)	11.0 (5.71)	19.5 (6.30)	**< 0.001**
**Mental Component Score**	45.4 (15.3)	51.6 (11.0)	36.5 (16.3)	**< 0.001**
**PHQ-2 Score**	0.986 (1.50)	0 (0)	2.41 (1.43)	**< 0.001**

Mean (SD) or count (percentage) are listed. P-values were calculated using chi-square (categorical variables), and two-sample t-test (parametric continuous variables). Significant p-values are bolded

n = 60 participants for Life’s Simple 7 Score; 61 participants for annual income and LS7 Diet; 70 participants for systolic and diastolic blood pressure and CES-D; and 71 participants for all other categories

^a^ Physical activity was calculated from the Centers for Medicare and Medicaid Services (CMS) Accountable Health Communities Health-Related Social Needs Screening Tool’s 2 questions on physical activity

Abbreviations: AHA = American Heart Association, LS7 = Life’s Simple 7, Cardiovascular Health recommendations were defined by AHA 2020 guidelines (Supplemental Table 4).

SI conversion factors: To convert total cholesterol from millimoles per liter to milligrams per deciliter, multiply by 38.6; to convert glucose from millimoles per liter to milligrams per deciliter, multiply by 18.

The change in mental health measures from baseline to 12 and 24 weeks are shown in Table 2. In fully adjusted analyses, at 12 and 24 weeks: (1) PHQ-2 decreased 0.43 (95%CI: -0.81, -0.06) and 0.41 (95%CI: -0.75, -0.07), respectively, from a baseline of 1.04 (95%CI: 0.65, 1.43); (2) CES-D scores decreased 2.12 (95%CI: -4.46, 0.22) and 2.70, 95%CI:-4.80, -0.60), respectively, from a baseline of 10.88 (95%CI: 8.33, 13.43) and; (3) PSS-10 decreased 1.80 (95%CI: -3.79, 0.19) and 1.73 (95%CI: -3.56, 0.10), respectively, from a baseline of 14.75 (95%CI: 12.48, 17.01). The SF-36 MCS score non-significantly increased at 12 and 24 weeks. The odds of CES-D ≥16 were numerically but not statistically significantly lower at 12 (OR 0.48, 95%CI: 0.16, 1.43) and 24 weeks (OR 0.57, 95%CI: 0.22, 1.49) in models adjusted for age.

**Table 2:** Longitudinal Change in Mental Health Measures at 12 and 24 weeks in Black Impact

Measure^a^	Time	Number	Unadjusted	95% CI	p-value	Adjusted for Age	95% CI	p-value	Adjusted for age, education, and income	95% CI	p-value
**Patient Health Questionnaire-2 (PHQ-2) (Range 0–6)**											
PHQ-2	Baseline	71	1.01	(0.71, 1.32)	.	1.01	(0.70, 1.32)	.	1.04	(0.65, 1.43)	.
PHQ-2	week12–baseline	41	-0.39	(-0.74, -0.05)	**0.027**	-0.39	(-0.74, -0.04)	**0.029**	-0.43	(-0.81, -0.06)	**0.023**
PHQ-2	week24–baseline	52	-0.29	(-0.60, 0.03)	0.076	-0.28	(-0.60, 0.03)	0.080	-0.41	(-0.75, -0.07)	**0.018**
**Center for Epidemiologic Studies Depression (CES-D) (Range 0–60)**											
CES-D Score	Baseline	70	10.91	(8.96, 12.87)	.	10.77	(8.84, 12.71)	.	10.88	(8.33, 13.43)	.
CES-D Score	week12–baseline	39	-2.03	(-4.14, 0.07)	0.058	-1.96	(-4.07, 0.14)	0.068	-2.12	(-4.46, 0.22)	0.076
CES-D Score	week24–baseline	51	-2.41	(-4.30, -0.51)	**0.014**	-2.32	(-4.22, -0.42)	**0.017**	-2.70	(-4.80, -0.60)	**0.012**
**Perceived Stress Scale 10 (PSS) (Range 0–40)**											
PSS-10	Baseline	71	14.45	(12.74, 16.16)	.	14.41	(12.69, 16.13)	.	14.75	(12.48, 17.01)	.
PSS-10	week12–baseline	42	-1.71	(-3.51, 0.09)	0.062	-1.69	(-3.49, 0.11)	0.066	-1.80	(-3.79, 0.19)	0.076
PSS-10	week24–baseline	52	-1.42	(-3.08, 0.24)	0.092	-1.40	(-3.06, 0.26)	0.097	-1.73	(-3.56, 0.10)	0.064
**36-item Short Form Survey (SF-36) Mental Component Score (MCS) (Range 0-100)**											
SF-36 MCS	Baseline	71	45.41	(42.14, 48.67)	.	45.57	(42.33, 48.80)	.	46.57	(42.29, 50.85)	.
SF-36 MCS	week12–baseline	42	1.97	(-2.11, 6.05)	0.342	1.86	(-2.23, 5.95)	0.369	0.98	(-3.50, 5.47)	0.664
SF-36 MCS	week24–baseline	52	1.42	(-2.35, 5.19)	0.456	1.30	(-2.48, 5.07)	0.498	1.53	(-2.60, 5.66)	0.464
**Measure** ^**b**^		**Time**	**Number**	**Number ≥ 16**	**Proportion**	**Unadjusted**	**95% CI**	**p-value**	**Adjusted for Age**	**95% CI**	**p-value**
Depressive Symptoms ≥ 16		Baseline	70	18	25.71%	.		.			
		week12–baseline	39	6	15.38%	0.47	(0.16, 1.40)	0.173	0.48	(0.16, 1.43)	0.185
		week24–baseline	51	9	17.65%	0.56	(0.21, 1.45)	0.228	0.57	(0.22, 1.49)	0.248

^a^ Statistical method, Linear mixed models were used to explore the change of outcome measures across time with random intercepts for each participant. Differences between each time point with baseline and 95% confidence intervals (CI) were reported

^b^ Statistical method, for binary outcomes: Generalized mixed models with random intercepts were used to explore the change of outcome measures across time. Models adjusted for age, education and income did not converge

Significant findings are bolded

In Table 3, a 1-point higher baseline CES-D was associated with less improvement in LS5 score at week 12 (-0.040, 95%CI: -0.076, -0.005) and 24 (-0.032, 95%CI: -0.067, 0.003). Similar findings were shown for PSS with a 1-point higher PSS associated with less improvement in LS5 score at week 12 (-0.040, 95%CI: -0.075, -0.005) and 24 (-0.034, 95%CI: -0.068, 0.001). There were no associations of mental health measures with change in LS6 or LS7 at weeks 12 or 24.

**Table 3:** The Relationship between Mental Health at Baseline and Change in Cardiovascular Health Scores

		Baseline Patient Health Questionnaire-2 (PHQ-2)				Baseline Center for Epidemiologic Studies Depression (CES-D)				Baseline 36-item Short Form Survey (SF-36) Mental Component Score (MCS)				Baseline Perceived Stress Scale-10				
outcome	covariate	Estimate	P-value	2.5%	97.5%	Estimate	P-value	2.5%	97.5%	Estimate	P-value	2.5%	97.5%	Estimate	P-value	2.5%	97.5%	
**LS5**	(Intercept)	**5.195**	**0.000**	**4.715**	**5.675**	**5.290**	**0.000**	**4.876**	**5.703**	**5.149**	**0.000**	**4.726**	**5.572**	**5.279**	**0.000**	**4.878**	**5.681**	
	Week 12	**0.695**	**0.001**	**0.312**	**1.077**	**0.639**	**0.000**	**0.312**	**0.965**	**0.641**	**0.000**	**0.299**	**0.983**	**0.544**	**0.001**	**0.229**	**0.860**	
	Week 24	**0.771**	**0.000**	**0.392**	**1.151**	**0.768**	**0.000**	**0.442**	**1.094**	**0.675**	**0.000**	**0.339**	**1.012**	**0.671**	**0.000**	**0.357**	**0.985**	
	LS5	0.094	0.498	-0.176	0.363	0.008	0.742	-0.037	0.052	-0.024	0.080	-0.050	0.002	0.022	0.435	-0.033	0.078	
	Week 12*LS5	-0.164	0.130	-0.374	0.046	**-0.040**	**0.028**	**-0.075**	**-0.005**	0.018	0.088	-0.002	0.038	-0.041	0.062	-0.084	0.001	
	Week 24*LS5	-0.114	0.294	-0.323	0.096	-0.034	0.063	-0.068	0.001	0.003	0.810	-0.018	0.023	-0.041	0.063	-0.083	0.001	
**LS6**	(Intercept)	**6.760**	**0.000**	**6.250**	**7.270**	**6.766**	**0.000**	**6.321**	**7.210**	**6.664**	**0.000**	**6.211**	**7.116**	**6.744**	**0.000**	**6.317**	**7.172**	
	Week 12	**0.661**	**0.007**	**0.196**	**1.125**	**0.640**	**0.002**	**0.240**	**1.039**	**0.612**	**0.005**	**0.197**	**1.027**	**0.578**	**0.004**	**0.193**	**0.964**	
	Week 24	**0.818**	**0.001**	**0.343**	**1.297**	**0.794**	**0.000**	**0.389**	**1.202**	**0.672**	**0.002**	**0.255**	**1.091**	**0.741**	**0.000**	**0.352**	**1.132**	
	LS6	-0.015	0.920	-0.301	0.271	-0.003	0.895	-0.051	0.045	-0.014	0.331	-0.042	0.014	0.005	0.857	-0.054	0.064	
	Week 12*LS6	-0.091	0.485	-0.342	0.162	-0.029	0.185	-0.071	0.013	0.007	0.579	-0.018	0.032	-0.027	0.314	-0.078	0.025	
	Week 24*LS6	-0.081	0.535	-0.335	0.172	-0.025	0.256	-0.067	0.017	-0.009	0.474	-0.034	0.015	-0.027	0.310	-0.079	0.025	
**LS7**	(Intercept)	**7.484**	**0.000**	**6.886**	**8.081**	**7.450**	**0.000**	**6.941**	**7.958**	**7.337**	**0.000**	**6.812**	**7.861**	**7.444**	**0.000**	**6.947**	**7.940**	
	Week 12	**0.711**	**0.016**	**0.152**	**1.269**	**0.773**	**0.003**	**0.291**	**1.257**	**0.652**	**0.015**	**0.145**	**1.160**	**0.692**	**0.005**	**0.230**	**1.157**	
	Week 24	**1.061**	**0.001**	**0.492**	**1.631**	**1.025**	**0.000**	**0.540**	**1.512**	**0.839**	**0.002**	**0.334**	**1.346**	**0.919**	**0.000**	**0.450**	**1.391**	
	LS7	-0.035	0.834	-0.356	0.286	0.000	0.995	-0.054	0.053	-0.016	0.296	-0.047	0.014	0.011	0.750	-0.055	0.077	
	Week 12*LS7	-0.025	0.870	-0.316	0.269	-0.029	0.231	-0.075	0.018	-0.005	0.738	-0.033	0.023	-0.034	0.256	-0.092	0.024	
	Week 24*LS7	-0.154	0.377	-0.488	0.183	-0.042	0.127	-0.095	0.011	-0.016	0.302	-0.047	0.014	-0.047	0.150	-0.110	0.016	

Intercept = Mean at baseline when the mental health measure is zero

Week 12 = Change in cardiovascular health score at week 12 when the mental health measure is held constant at zero

Week 24 = Change in cardiovascular health score at week 24 when the mental health measure is held constant at zero

Life’s Simple 7 = A 1-unit increase in mental health measure corresponds to the effect estimate change in cardiovascular health score at baseline

Week 12*LS7 = A 1-unit higher mental health measure at baseline is associated with an effect estimate change in cardiovascular health score at week 12

Week 24*LS7 = A 1-unit higher mental health measure at baseline is associated with an effect estimate change in cardiovascular health score at week 24

**Interpretation Example**:

The mean LS5 score at baseline when the perceived stress score is zero is 4.94 (95%CI: 4.04, 5.84)

Change in LS5 at week 12 when the perceived stress score is held constant at zero is 1.13 points (95%CI: 0.44, 1.82)

Change in LS5 at week 24 when the perceived stress score is held constant at zero is 1.25 points (95%CI: 0.57, 1.93)

A 1-unit increase in perceived stress score corresponds to a non-significant 0.03-point higher (0.03, 95%CI: -0.03, 0.08) LS5 score at baseline

A 1-unit higher perceived stress score at baseline is associated with a -0.04-unit decrease (-0.04, 95%CI: -0.09, 0.00) LS5 score at week 12

A 1-unit higher perceived stress score at baseline is associated with a -0.04-unit decrease (-0.04, 95%CI: -0.09, 0.00) LS5 score at week 24

**Overall interpretation**: There are trends towards higher baseline CES-D and perceived stress scores being associated with change in LS5 measures at week 12 and 24. There were no associations of mental health measures with change in LS6 or LS7 at weeks 12 or 24.

Significant findings are bolded.

In Table 4 and Supplemental Table 3, the comparison of change in mental health measures with change in cardiovascular health scores was evaluated. The longitudinal change in mental health scores (CES-D, PHQ-2, PSS, SF-36 MCS) were significantly associated with each other (p < 0.05). There was no longitudinal association of mental health scores with cardiovascular health scores (LS5, LS6 and LS7).

**Table 4:** Comparison of Change in Mental Health Measures with Change in Cardiovascular Health Scores using Linear Regression Models with Mixed Effects Generated Slopes of Mental Health Measures and Cardiovascular Health Scores across the study (Baseline, Week 12, and Week 24)

measure	CESD	PHQ2	LS5	LS6C	LS7C	MCS	PSS
CESD		**5.954 (< 0.001)**	4.099 (0.148)	0.804 (0.597)	0.303 (0.747)	**-0.469 (0.001)**	**0.751 (< 0.001)**
PHQ2	**0.081 (< 0.001)**		0.079 (0.808)	0.007 (0.968)	0.018 (0.870)	**-0.078 (< 0.001)**	**0.092 (< 0.001)**
LS5	0.007 (0.148)	0.011 (0.808)		**0.448 (< 0.001)**	**0.211 (< 0.001)**	-0.001 (0.860)	-0.001 (0.906)
LS6C	0.005 (0.597)	0.003 (0.968)	**1.508 (< 0.001)**		**0.505 (< 0.001)**	0.003 (0.802)	-0.015 (0.272)
LS7C	0.006 (0.747)	0.026 (0.870)	**2.250 (< 0.001)**	**1.611 (< 0.001)**		0.012 (0.600)	-0.027 (0.297)
MCS	**-0.297 (0.001)**	**-3.597 (< 0.001)**	-0.388 (0.860)	0.302 (0.802)	0.391 (0.600)		**-0.347 (0.009)**
PSS	**0.380 (< 0.001)**	**3.393 (< 0.001)**	-0.232 (0.906)	-1.174 (0.272)	-0.688 (0.297)	**-0.276 (0.009)**	

The above numbers were calculated by fitting a mixed effect model for each outcome variable. The column names are the independent variables and the row names are the dependent variables. The mixed effect models had a random slope for time for each participant. These random slopes were extracted and saved. Then, linear models were run comparing the generated slopes for the independent variables to the slopes from the dependent variable. The numbers above are estimates from the linear models. The numbers in parentheses are p-values rounded to three decimal places

CESD: Center for Epidemiological Studies Depression Scale [46, 71]. PHQ2: Patient Health Questionnaire 2-question depression screener. LS5: Life Simple 7 score that does not include diet and physical activity (0–10). LS6C: Life Simple 7 score that does not include diet (0–12). The C denotes physical activity was calculated based on the CMS physical activity score or weekly reported physical activity minutes if CMS physical activity was missing. LS7C: Life Simple 7 score (0–14). The C denotes physical activity was calculated based on the CMS physical activity score or weekly reported physical activity minutes if CMS physical activity was missing. MCS: Mental component score of the SF-36. PSS: Perceived Stress Scale score

Some of the mixed effects models were singular when generating the slopes. Even so, the results are as expected (LS7 variables correlate with LS7 variables, and mental health variables correlate with mental health variables). We performed a different analysis to answer the same question to verify the results from the above analysis (see below)

Interpretation example: A 1-unit change in CES-D slope is associated with a 0.079 unit change in PHQ-2 slope (p < 0.001), where the slopes are the linear change in score over all time points.

Overall interpretation: There is no association of change in mental health measures with change in cardiovascular health scores. The change in one mental health measure was associated with the change in other mental health measures.

Significant findings are bolded.

## Discussion

Black Impact, a novel 24-week community-based lifestyle intervention focused on physical activity and health education in Black men, demonstrated improvements in mental health, including reductions in depressive scores and perceived stress. While there was no association of baseline mental health measures with baseline overall cardiovascular health scores, higher baseline depressive symptoms and perceived stress were associated lower improvements in cardiovascular health scores inclusive of blood pressure, cholesterol, glucose, body mass index and smoking, over 12 and 24 weeks. The change in mental health measures did not influence the change in cardiovascular health scores during the intervention. Limited data exist on interventions to improve LS7 overall in Black Americans, with only two published studies focusing on all 7 components prior to Black Impact [22, 54, 55]. Both of these studies were in majority Black women and neither evaluated mental health as an outcome [22, 54, 55]. Thus, Black Impact is the first LS7-based intervention to show improvements in mental health among Black Men. Given the burden of poor physical and mental health in Black men, Black Impact provides support for larger, randomized trials to test interventions focused on improving mental and physical health using the LS7 framework.

### The association of mental health measures with life’s simple 7 in black men

Divergent from the baseline mental health to LS7 associations in Black Impact, the majority of the extant literature in multi-racial and Black American observational cohort studies demonstrates that depressive symptoms and perceived stress are associated with poor levels of LS7 scores with an overall greater effect among the behavioral components of LS7 (smoking, physical activity, diet, and body mass index) including among Black Americans in the REGARDS study [19, 38, 41]. In Black Americans in the Jackson Heart Study, participants with higher scores for minor stressors and stressful major life events were less likely to achieve higher levels of CVH scores, with no difference between men and women [34]; and participants with hypertension with both high stress and depressive symptoms had lower composite LS7 than those with low stress and depressive symptoms [56]. While the findings are not consistent with the cross-sectional Black impact results, they are consistent with longitudinal findings in Black Impact that baseline depression was associated with a significant reduction in improvement for LS5 at Week 12 and trends towards reductions at Week 24 for depressive symptoms and Week 12 and 24 for perceived stress.

Additionally, the mental health-LS7 relationship is bi-directional with observational studies showing an association of LS7 with depressive symptoms and stress [35, 39, 57]. Baseline behavioral CVH score was inversely associated with perceived stress at four years, even after adjustment for perceived stress measured at baseline (p < 0.001) [41]. The differential findings in Black Impact may be due to a smaller sample size than prior studies, although it is important to note that the majority of the extant literature does not specifically examine these relationships among Black men. Among Black men, there may be domains of depressive or stress symptoms that are not captured by CES-D, PHQ-2, MCS or PSS. In a recent study by Adams et al. [58], investigators hypothesized that “Black men’s marginalized social status in the United States fundamentally shapes their depression symptoms, and ultimately, the ways in which they conceptualize the depression experience”. Concept mapping, a structured mixed methods approach, to characterize depressive symptoms in a community-based sample of Black men can be used to identify clusters of previously identified items including social pressures that are not captured in the validated measures of depression and stress used in Black Impact and other studies.

Thus, further work to contextualize the bi-directional association of mental health and Life’s Simple 7 is warranted in Black men. Additionally, delineating the underlying mechanistic pathways that mediate the mental health-cardiovascular risk-cardiovascular outcomes pathway is pivotal, including the role of allostatic load. Our group has previously shown that the neuroendocrine allostatic load subsystems (cortisol, aldosterone) and overall allostatic load (metabolic, cardiovascular, immune and neuroendocrine) are associated with coronary heart disease in Black men [12].

### Black impact mental health improvement effect size

The Black Impact intervention was associated with a 25% reduction in CES-D scores over 24 weeks in a sample of individuals with low levels of depressive symptoms at baseline (mean 10.9 [SD 9.0]). By comparison in a culturally-adapted depression intervention for African American men and women experiencing depression, CES-D-measured depressive symptoms decreased by 43% over 6 months from a higher baseline (mean 26.9 [SD 9.6]) [59, 60]. In the intervention, participants met for 12 weeks for 2.5 h per week for cognitive behavioral therapy (CBT) and psychoeducation facilitated by African American Master’s level counselors. In a standard and patient-centered, culturally-tailored collaborative care (CC) intervention for African American patients with major depressive disorder (MDD), delivered by a primary care physician and consultation-liaison psychiatrist team that focused on education and evidenced-based practice [61], CES-D scores improved from 29.84 to 30.17 in standard and in patient-centered groups from 20.64 to 22.67, representing decreases of ~ 31% and 25%, respectively at 6 months. Thus, the effect size seen in Black Impact is lower than the CBT-focused intervention and consistent with improvements seen in the primary care led intervention [61].

### Potential components of black impact leading to improvements in mental health

Many factors of the Black Impact Program may have led to improvements in mental health: the program included two sessions that specifically addressed mental health, fourteen of the men participated in an additional two-hour session to discuss mental health in a community large group format (100–200 men), the program promoted physical activity with 45 min of physical activity in community parks per week, the men in the program built a camaraderie over the course of the intervention and were paired in teams influencing social networks and potentially decreasing isolation. These components are supported by the “Clinical guidelines for the use of lifestyle-based mental health care in major depressive disorder” [62]. We will discuss two of these components in further detail.

### Physical activity

Among many racial/ethnic groups, a recent meta-analysis revealed the anti-depressive effect of exercise, even when adjusting for publication bias [28]. Forty-nine prospective studies (n = 266,939) across the world show a 22% and 21% lower odds of incident depression in adults and elderly persons, respectively, with high vs. low levels of physical activity [28]. In Black American adults, a systematic review of 13 randomized controlled trials, showed that while there was an effect of increasing physical activity in reducing depressive symptoms in Black adults, the majority of the studies analyzed were in Black women [29]. In a recent pilot RCT, resistance training improved depressive symptoms to a greater extent than health, wellness and education in a pilot of Black men over 12 weeks [63]. Consistent with these findings, in Black Impact at baseline, individuals with a PHQ-2 score of 0 vs. ≥1 had higher levels of physical activity.

Recently, there has been greater recognition of the additional benefits of “green exercise”, being physically active in the natural environment, on mental health with the greatest benefit among individuals with lower levels of mental health [62, 64]. Green exercise may lower negative affect, including anxiety, tension, anger, depression, and fatigue [65]. Nature-based interventions were effective for improving depressive mood, reducing anxiety, improving positive affect, and reducing negative affect. The most effective interventions were offered for between 8 and 12 weeks, and the optimal dose ranged from 20 to 90 min [66]. Linking back to allostatic load, spending time in nature also improves cortisol parameters [67], and cortisol dysregulation is a key underlying mechanism linking stress and depression with chronic disease [68]. The workouts for Black Impact were completed in urban parks with green spaces and large tree canopies. Thus, the Black Impact physical activity regimen, particularly in the natural environment, may have contributed to the improvements in mental health, and future studies should include objective measures of physical activity and include larger representative samples of Black men in a randomized intervention to further delineate the contribution of physical activity to improvements in mental health in Black Impact.

### Social networks

Social networks are the social ties that link people together through communication [30]. Two dimensions of social networks are social support and social connectedness. Social support is defined as the frequency or number of contacts a person has with friends, family members, and other supportive network members. Social connectedness refers to: (1) the structural, functional, and qualitative aspects of social relationships, including social isolation and loneliness [31, 32]; and (2) the strength or closeness of ties adults experience through friendships, both casual and intimate [33]. Social support and connectedness are often cited as a buffers in the relationship between stress and depression [33]. Previous research on social support has found that increased positive social support leads to a decrease in depressive symptoms and that social support acts as a buffer against stress [33]. Increasing social connectedness has been shown to reduce depression in underserved older adults living with depression in a multi-racial/ethnic majority female sample [32]. The organization of the men into teams was purposeful to induce a sense of camaraderie and to build social networks to enhance social support and social connectedness. In a survey of community-dwelling Black Americans, Black men were less open to acknowledging psychological problems and seeking help compared to women and were very concerned about stigma associated with mental health. Both men and women preferred religious coping and informal support networks over professional help and seeking mental health services [69]. Thus, the building and enhancement of social networks may be another potential factor that led to improvements in mental health in Black Impact and is an area of future quantitative and qualitative exploration in larger studies.

### Strengths/limitations

The strengths of our study include: (1) a focus on an understudied population with significant disparities in mental and cardiovascular health; (2) utilization of a community engagement framework for the community-based participatory research (CBPR) that addressed mental health needs in Black Impact; (3) the use of validated surveys to assess mental health; and (4) biometric data collection using evidenced-based approaches including collection by trained health professionals. Despite these strengths, the study should be considered in light of some limitations. As we have noted previously [27, 45], the study was not randomized due to: (1) no previous test of intervention feasibility and acceptability; and (2) concerns raised from community members in regards to not receiving a potentially beneficial intervention. A second limitation is the lack of a control group [27, 45]. Third, Black Impact participants may not be representative of other populations of Black men and did not have high levels of depressive symptoms at baseline. Fourth, data was not collected on previous diagnoses of depression or anti-depressant medications, which would be helpful in determining improvements in mental health among subpopulations. Lastly, our study was performed during the COVID-19 pandemic, which may have influenced improvements in mental health due to higher levels of social isolation in the general population during the COVID-19 pandemic. Thus, the increased social connectedness and physical activity may have had enhanced effects due to the general isolation experienced during the COVID-19 pandemic. Future larger randomized studies are planned to address these limitations.

## Conclusion

Efficacious interventions that improve mental health and physical health in Black men are urgently needed to close disparities in mental and physical health that lead to vast inequities in life expectancy. To our knowledge, Black Impact is the first intervention to show improvements in mental health in a comprehensive community team-based physical activity, health education and social needs intervention among Black men, providing a potential novel comprehensive approach to improving mental health in Black men. The findings yield further support for the recent guidelines for the use of lifestyle-based mental health care through the use of physical activity and exercise, sleep, diet, green space, smoking cessation and loneliness and social support, which are all aligned with the Black Impact intervention and the AHA CVH conceptualization, particularly with the addition of sleep in Life’s Essential 8 [62, 70]. The lifestyle-based mental health care guidelines also note that implementation considerations include the need for support networks and the importance of partnering such recommendations with behavior change support, and intervention delivery using a biopsychosocial-cultural framework [62], all critical components of Black Impact. Black Impact should be tested in a larger, randomized controlled interventions to examine efficacy and to further explore the underlying mechanisms driving improvements in mental health among Black men.

### Electronic supplementary material

Below is the link to the electronic supplementary material.

**Supplementary Material 1**: Supplemental Tables 1–4

**Supplementary Material 2**: Supplemental Figure 1

## Data Availability

The datasets generated and/or analyzed during the current study are not publicly available due to agreements made with participants through the informed consent form and outlined procedures for data-handling therein. Anonymized data are available from the corresponding author on reasonable request.
