# Supplementary material for: Improving mental health in black men through a 24-week community-based lifestyle change intervention: the black impact program
Source: BMC Psychiatry. 2024 Jan 9;24:34. doi: 10.1186/s12888-023-05064-5 (PMC10775551; doi:10.1186/s12888-023-05064-5)
Supplement: Supplementary file 1 — Supplementary Material 1: Supplemental Tables 1–4 [file 12888_2023_5064_MOESM1_ESM.docx]

**Supplemental Table 1.** Baseline Continuous Correlations between Mental Health Measures and Cardiovascular Health Scores

| **measure** | **CES-D** | **PHQ-2** | **LS5** | **LS6C** | **LS7C** | **MCS** | **PSS** |
| --- | --- | --- | --- | --- | --- | --- | --- |
| CES-D |  | 4.853 (<0.001) | 0.29 (0.655) | -0.019 (0.975) | 0.086 (0.902) | -0.384 (<0.001) | 0.947 (<0.001) |
| PHQ-2 | 0.136 (<0.001) |  | 0.084 (0.435) | -0.002 (0.983) | -0.016 (0.89) | -0.06 (<0.001) | 0.142 (<0.001) |
| LS5 | 0.01 (0.655) | 0.107 (0.435) |  | 0.907 (<0.001) | 0.822 (<0.001) | -0.024 (0.065) | 0.026 (0.361) |
| LS6C | -0.001 (0.975) | -0.003 (0.983) | 0.959 (<0.001) |  | 0.904 (<0.001) | -0.015 (0.286) | 0.009 (0.765) |
| LS7C | 0.003 (0.902) | -0.021 (0.89) | 0.936 (<0.001) | 1.005 (<0.001) |  | -0.017 (0.23) | 0.015 (0.627) |
| MCS | -1.118 (<0.001) | -6.18 (<0.001) | -2.013 (0.065) | -1.143 (0.286) | -1.447 (0.23) |  | -1.469 (<0.001) |
| PSS | 0.63 (<0.001) | 3.353 (<0.001) | 0.478 (0.361) | 0.152 (0.765) | 0.276 (0.627) | -0.334 (<0.001) |  |

**Legend Supplemental Table 1**. The estimates were generated from unadjusted linear models fit on baseline values of outcome variables. Numbers in parentheses are p-values rounded to 3 decimal places. The column names are the independent variables and the row names are the dependent variables.

**Interpretation Example:** For a 1-point increase in CES-D score at baseline there is a 0.136 point increase in PHQ-2 score at baseline.

CES-D: Center for Epidemiological Studies Depression Scale. PHQ-2: Patient Health Questionnaire 2-question depression screener. LS5: Life Simple 7 score that does not include diet and physical activity (0-10). LS6C: Life Simple 7 score that does not include diet (0-12). The C denotes physical activity was calculated based on the CMS physical activity score or weekly reported physical activity minutes if CMS physical activity was missing. LS7C: Life Simple 7 score (0-14). The C denotes physical activity was calculated based on the CMS physical activity score or weekly reported physical activity minutes if CMS physical activity was missing. MCS: Mental component score of the SF-36. PSS: Perceived Stress Scale score.

**Overall Interpretation:** There is no significant correlation of baseline mental health measures with baseline cardiovascular health scores. There are correlations among the various mental health measures (CES-D, PHQ-2, MCS of SF-36 and PSS).

**Supplemental Table 2**. Pearson correlation coefficients between baseline mental health and cardiovascular health components

| **measure** | **CES-D** | **PHQ-2** | **LS5** | **LS6C** | **LS7C** | **MCS** | **PSS** |
| --- | --- | --- | --- | --- | --- | --- | --- |
| CES-D |  | 0.812 | 0.055 | -0.004 | 0.016 | -0.655 | 0.773 |
| PHQ-2 | 0.812 |  | 0.095 | -0.003 | -0.018 | -0.607 | 0.690 |
| LS5 | 0.055 | 0.095 |  | 0.933 | 0.877 | -0.222 | 0.111 |
| LS6C | -0.004 | -0.003 | 0.933 |  | 0.953 | -0.129 | 0.036 |
| LS7C | 0.016 | -0.018 | 0.877 | 0.953 |  | -0.157 | 0.064 |
| MCS | -0.655 | -0.607 | -0.222 | -0.129 | -0.157 |  | -0.701 |
| PSS | 0.773 | 0.690 | 0.111 | 0.036 | 0.064 | -0.701 |  |

**Legend Supplemental Table 2:**

**Interpretation:**

| **Absolute Magnitude of Correlation Coefficient** | **Interpretation** |
| --- | --- |
| 0.00-0.10 | Negligible Correlation |
| 0.10-0.39 | Weak Correlation |
| 0.40-0.69 | Moderate Correlation |
| 0.70-0.89 | Strong Correlation |
| 0.90-1.00 | Very Strong Correlation |

Abbreviations: CES-D: Center for Epidemiological Studies Depression Scale. PHQ-2: Patient Health Questionnaire 2-question depression screener. LS5: Life Simple 7 score that does not include diet and physical activity (0-10). LS6C: Life Simple 7 score that does not include diet (0-12). The C denotes physical activity was calculated based on the CMS physical activity score or weekly reported physical activity minutes if CMS physical activity was missing. LS7C: Life Simple 7 score (0-14). The C denotes physical activity was calculated based on the CMS physical activity score or weekly reported physical activity minutes if CMS physical activity was missing. MCS: Mental component score of the SF-36. PSS: Perceived Stress Scale score.

**Supplemental Table 3.** Comparison of Change in Depressive Symptom Measures with Change in Cardiovascular Health Scores using a Difference versus Difference Linear Regression Mode

| **Measure** | **CES-D12** | **CES-D24** | **PHQ2.12** | **PHQ2.24** | **LS5.12** | **LS5.24** | **LS6C12** | **LS6C24** | **LS7C12** | **LS7C24** | **MCS12** | **MCS24** | **PSS12** | **PSS24** |
| --- | --- | --- | --- | --- | --- | --- | --- | --- | --- | --- | --- | --- | --- | --- |
| CES-D12 |  | 0.63 (<0.001) | 2.406 (0.007) | 1.533 (0.118) | 0.535 (0.511) | 0.91 (0.346) | 0.353 (0.606) | 0.575 (0.457) | -0.223 (0.765) | 0.52 (0.504) | -0.164 (0.018) | -0.036 (0.667) | 0.751 (<0.001) | 0.173 (0.329) |
| CES-D24 | 0.847 (<0.001) |  | 1.732 (0.125) | 3.991 (<0.001) | 2.14 (0.008) | 1.562 (0.065) | 1.652 (0.025) | 0.898 (0.213) | 1.273 (0.148) | 0.393 (0.555) | -0.137 (0.072) | -0.351 (<0.001) | 0.657 (0.004) | 0.611 (<0.001) |
| PHQ-2.12 | 0.074 (0.007) | 0.038 (0.125) |  | 0.662 (<0.001) | -0.006 (0.968) | -0.208 (0.225) | 0.035 (0.775) | -0.083 (0.557) | -0.014 (0.882) | 0.031 (0.768) | -0.006 (0.632) | -0.017 (0.222) | 0.064 (0.086) | 0.046 (0.111) |
| PHQ-2.24 | 0.047 (0.118) | 0.1 (<0.001) | 0.69 (<0.001) |  | 0.148 (0.266) | 0.028 (0.836) | 0.139 (0.245) | -0.015 (0.895) | 0.107 (0.399) | -0.127 (0.149) | 0.012 (0.305) | -0.058 (<0.001) | 0.027 (0.448) | 0.117 (<0.001) |
| LS5.12 | 0.023 (0.511) | 0.065 (0.008) | -0.008 (0.968) | 0.174 (0.266) |  | 0.707 (<0.001) | 0.749 (<0.001) | 0.518 (<0.001) | 0.724 (<0.001) | 0.353 (0.004) | 0.006 (0.676) | -0.018 (0.193) | 0.041 (0.351) | 0.043 (0.125) |
| LS5.24 | 0.027 (0.346) | 0.044 (0.065) | -0.185 (0.225) | 0.031 (0.836) | 0.641 (<0.001) |  | 0.439 (<0.001) | 0.773 (<0.001) | 0.507 (<0.001) | 0.633 (<0.001) | 0.007 (0.539) | 0.012 (0.359) | 0.016 (0.65) | 0.012 (0.653) |
| LS6C12 | 0.022 (0.606) | 0.065 (0.025) | 0.063 (0.775) | 0.21 (0.245) | 1.05 (<0.001) | 0.678 (<0.001) |  | 0.633 (<0.001) | 0.939 (<0.001) | 0.478 (0.001) | 0.012 (0.495) | -0.015 (0.367) | -0.014 (0.781) | 0.033 (0.32) |
| LS6C24 | 0.028 (0.457) | 0.036 (0.213) | -0.117 (0.557) | -0.023 (0.895) | 0.643 (<0.001) | 1.021 (<0.001) | 0.591 (<0.001) |  | 0.684 (<0.001) | 0.879 (<0.001) | 0.01 (0.518) | 0.026 (0.111) | -0.017 (0.727) | -0.021 (0.516) |
| LS7C12 | -0.015 (0.765) | 0.044 (0.148) | -0.056 (0.882) | 0.176 (0.399) | 0.963 (<0.001) | 0.786 (<0.001) | 0.949 (<0.001) | 0.712 (<0.001) |  | 0.626 (<0.001) | 0.034 (0.127) | -0.005 (0.797) | -0.053 (0.378) | 0.018 (0.595) |
| LS7C24 | 0.03 (0.504) | 0.024 (0.555) | 0.095 (0.768) | -0.438 (0.149) | 0.556 (0.004) | 1.117 (<0.001) | 0.561 (0.001) | 1.001 (<0.001) | 0.707 (<0.001) |  | 0.015 (0.455) | 0.045 (0.026) | -0.008 (0.884) | -0.055 (0.176) |
| MCS12 | -0.876 (0.018) | -0.651 (0.072) | -0.991 (0.632) | 2.495 (0.305) | 0.751 (0.676) | 1.308 (0.539) | 1.05 (0.495) | 1.138 (0.518) | 2.272 (0.127) | 1.25 (0.455) |  | 0.639 (0.001) | -1.505 (0.001) | -0.755 (0.077) |
| MCS24 | -0.156 (0.667) | -1.075 (<0.001) | -2.515 (0.222) | -7.014 (<0.001) | -1.899 (0.193) | 1.376 (0.359) | -1.155 (0.367) | 2.005 (0.111) | -0.374 (0.797) | 2.823 (0.026) | 0.443 (0.001) |  | -1.206 (0.003) | -1.474 (<0.001) |
| PSS12 | 0.432 (<0.001) | 0.329 (0.004) | 1.15 (0.086) | 0.601 (0.448) | 0.56 (0.351) | 0.324 (0.65) | -0.144 (0.781) | -0.205 (0.727) | -0.511 (0.378) | -0.089 (0.884) | -0.163 (0.001) | -0.184 (0.003) |  | 0.416 (0.002) |
| PSS24 | 0.167 (0.329) | 0.492 (<0.001) | 1.529 (0.111) | 3.646 (<0.001) | 1.134 (0.125) | 0.344 (0.653) | 0.66 (0.32) | -0.419 (0.516) | 0.407 (0.595) | -0.884 (0.176) | -0.115 (0.077) | -0.38 (<0.001) | 0.601 (0.002) |  |

**Legend Supplemental Table 3:**

The column names are the independent variables and the row names are the dependent variables.

The above numbers were calculated by subtracting the values of the outcome variables at each post-baseline time point and the baseline values of the same outcome variable for each participant. Then a linear model was fit where the differences in one outcome variable at one time point describe the differences in another outcome variable at one time point.

Names ending in 12 are week 12 differences; names ending in 24 are week 24 differences.

Numbers are estimates from linear models on the association between the indicated change scores (differences). Numbers in parentheses are p-values rounded to 3 decimal places.

CES-D: Center for Epidemiological Studies Depression Scale. PHQ-2: Patient Health Questionnaire 2-question depression screener. LS5: Life Simple 7 score that does not include diet and physical activity (0-10). LS6C: Life Simple 7 score that does not include diet (0-12). The C denotes physical activity was calculated based on the CMS physical activity score or weekly reported physical activity minutes if CMS physical activity was missing. LS7C: Life Simple 7 score (0-14). The C denotes physical activity was calculated based on the CMS physical activity score or weekly reported physical activity minutes if CMS physical activity was missing. MCS: Mental component score of the SF-36. PSS: Perceived Stress Scale score.

**Interpretation Example:** A 1-unit change in the difference between CES-D at Week 24 and baseline is associated with a 0.044 unit change in the difference between LS5 at Week 24 versus baseline (p=0.069).

**Overall Interpretation:** There is no association of change in mental health measures with change in cardiovascular health scores, although we did see significant association between CES-D at week 24 and LS5 at Week 12. The change in one mental health measure was associated with the change in other mental health measures.

**Supplemental Table 4.** American Heart Association Definitions of Poor, Intermediate, and Ideal Cardiovascular Health^a^

| Goal/Metric | Poor health | Intermediate health | Ideal health |
| --- | --- | --- | --- |
| Current smoking | Yes | Former ≤ 12 months | Never or quit >12 months |
| Total cholesterol | ≥ 240 mg/dl  (≥ 6.2 mmol/L) | 200-239 mg/dl  or treated to goal  (5.18-6.19 mmol/L) | <200 mg/dl  (<6.19 mmol/L) |
| Blood pressure | SBP ≥140  or  DBP ≥90 mmHg | SBP 120-139  or DBP 80-89 mmHg  or treated to goal | <120/<80 mmHg |
| Body mass index | ≥30 kg/m^2^ | 25-29.9 kg/m^2^ | <25 kg/m^2^ |
| Physical activity | None | 1–149 min/wk moderate intensity  or  1–74 min/wk vigorous intensity | ≥150 min/wk moderate intensity  or  ≥ 75 min/wk vigorous intensity |
| Healthy diet score^b^ | 0-1 components | 2-3 components | 4-5 components |
| Fasting glucose | ≥126 mg/dl  (≥7.00 mmol/L) | 100-125 mg/dl  or treated to goal  (5.55-6.99 mmol/L) | <100 mg/dl  (<5.55 mmol/L) |

Supplemental Table 4 Legend:

^a^ Adapted from The American Heart Association’s Strategic Planning Task Force and Statistical Committee 2020 Guidelines[17]

^b^ Adapted from The American Heart Association’s Strategic Planning Task Force and Statistical Committee 2020 Guidelines: Fruits and vegetables ≥4·5 cups/day, fish ≥two 3.5 ounce servings per week (non-fried), fiber-rich whole grains ≥ three 1 ounce-equivalent servings/day, sodium <1500 mg/day, and sugar-sweetened beverages ≤ 1884 kJ (36 ounces)/week.
