# Supplementary figures and images for: Improving mental health in black men through a 24-week community-based lifestyle change intervention: the black impact program
Source: BMC Psychiatry. 2024 Jan 9;24:34. doi: 10.1186/s12888-023-05064-5 (PMC10775551; doi:10.1186/s12888-023-05064-5)

Supplemental Figure 1. Study Consort Diagram

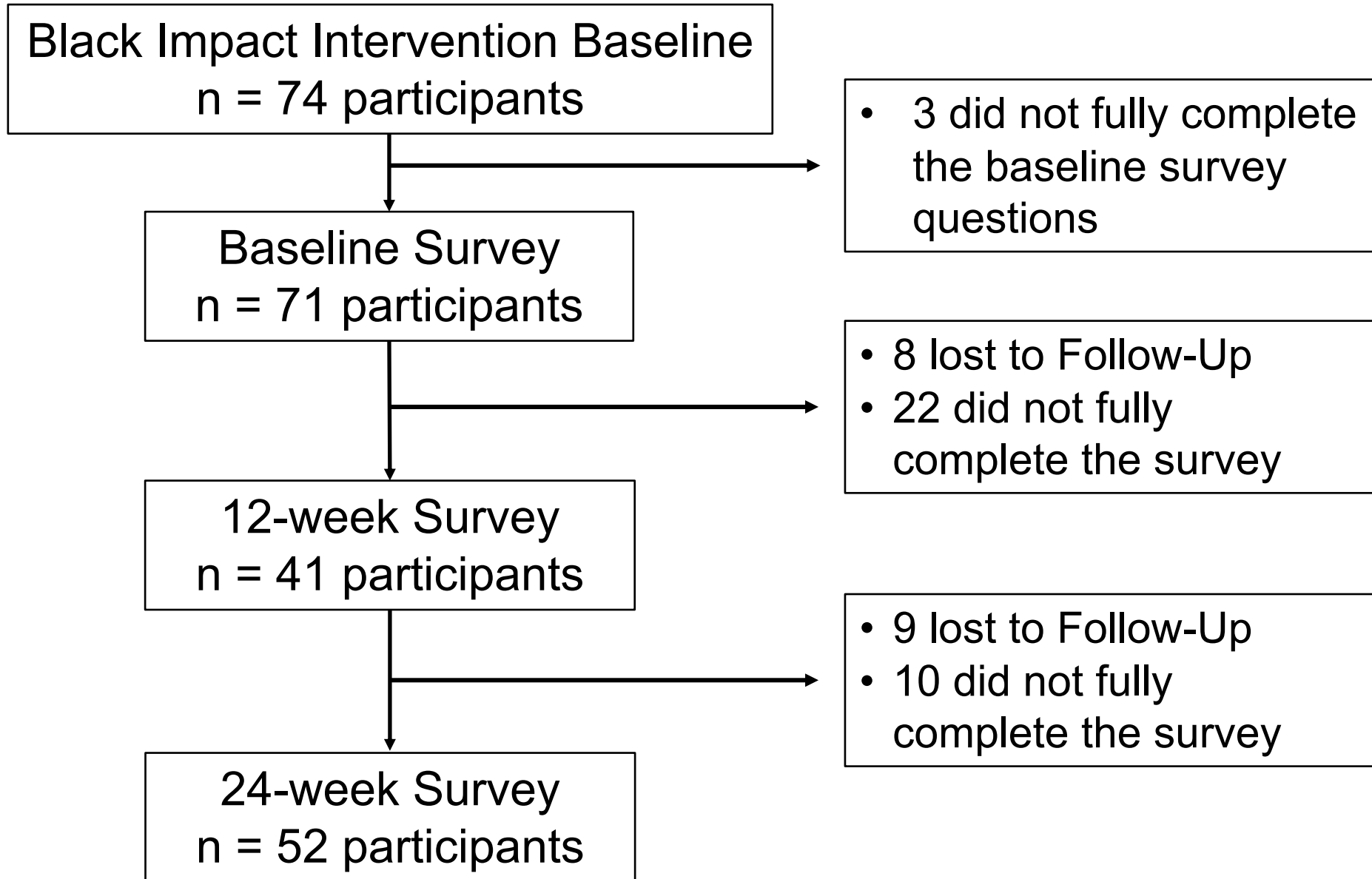

Supplement: Supplementary file 2 — Supplementary Material 2: Supplemental Figure 1 [file 12888_2023_5064_MOESM2_ESM.pdf]
